# Image Segmentation of the Ventricular Septum in Fetal Cardiac Ultrasound Videos Based on Deep Learning Using Time-Series Information

**DOI:** 10.3390/biom10111526

**Published:** 2020-11-08

**Authors:** Ai Dozen, Masaaki Komatsu, Akira Sakai, Reina Komatsu, Kanto Shozu, Hidenori Machino, Suguru Yasutomi, Tatsuya Arakaki, Ken Asada, Syuzo Kaneko, Ryu Matsuoka, Daisuke Aoki, Akihiko Sekizawa, Ryuji Hamamoto

**Affiliations:** 1 Division of Molecular Modification and Cancer Biology, National Cancer Center Research Institute, 5-1-1 Tsukiji, Chuo-ku, Tokyo 104-0045, Japan; adozen@ncc.go.jp (A.D.); kshozu@ncc.go.jp (K.S.); hidenori.machino@riken.jp (H.M.); ken.asada@riken.jp (K.A.); sykaneko@ncc.go.jp (S.K.); 2 Department of Obstetrics and Gynecology, Keio University School of Medicine, 35 Shinanomachi, Shinjuku-ku, Tokyo 160-8582, Japan; aoki@z7.keio.jp; 3 Cancer Translational Research Team, RIKEN Center for Advanced Intelligence Project, 1-4-1 Nihonbashi, Chuo-ku, Tokyo 103-0027, Japan; 4 Artificial Intelligence Laboratory, Fujitsu Laboratories Ltd., 4-1-1 Kamikodanaka, Nakahara-ku, Kawasaki, Kanagawa 211-8588, Japan; akira.sakai@fujitsu.com (A.S.); yasutomi.suguru@fujitsu.com (S.Y.); 5 RIKEN AIP-Fujitsu Collaboration Center, RIKEN Center for Advanced Intelligence Project, 1-4-1 Nihonbashi, Chuo-ku, Tokyo 103-0027, Japan; rkomatsu@med.showa-u.ac.jp (R.K.); ryu@med.showa-u.ac.jp (R.M.); 6 Biomedical Science and Engineering Track, Graduate School of Medical and Dental Sciences, Tokyo Medical and Dental University, 1-5-45 Yushima, Bunkyo-ku, Tokyo 113-8510, Japan; 7 Department of Obstetrics and Gynecology, Showa University School of Medicine, 1-5-8 Hatanodai, Shinagawa-ku, Tokyo 142-8666, Japan; arakakit@med.showa-u.ac.jp (T.A.); sekizawa@med.showa-u.ac.jp (A.S.)

**Keywords:** congenital heart disease, fetal cardiac ultrasound video, deep learning, segmentation, ventricular septum

## Abstract

Image segmentation is the pixel-by-pixel detection of objects, which is the most challenging but informative in the fundamental tasks of machine learning including image classification and object detection. Pixel-by-pixel segmentation is required to apply machine learning to support fetal cardiac ultrasound screening; we have to detect cardiac substructures precisely which are small and change shapes dynamically with fetal heartbeats, such as the ventricular septum. This task is difficult for general segmentation methods such as DeepLab v3+, and U-net. Hence, here we proposed a novel segmentation method named Cropping-Segmentation-Calibration (CSC) that is specific to the ventricular septum in ultrasound videos in this study. CSC employs the time-series information of videos and specific section information to calibrate the output of U-net. The actual sections of the ventricular septum were annotated in 615 frames from 421 normal fetal cardiac ultrasound videos of 211 pregnant women who were screened. The dataset was assigned a ratio of 2:1, which corresponded to a ratio of the training to test data, and three-fold cross-validation was conducted. The segmentation results of DeepLab v3+, U-net, and CSC were evaluated using the values of the mean intersection over union (mIoU), which were 0.0224, 0.1519, and 0.5543, respectively. The results reveal the superior performance of CSC.

## 1. Introduction

Congenital heart disease (CHD) is the most frequent congenital morphological abnormality, occurring in approximately 1% of newborns and accounting for 20–40% of neonatal deaths attributable to congenital diseases [1,2,3,4,5]. CHDs are related to abnormal pregnancy outcomes such as preeclampsia, small for gestational age, placental disorders, and preterm birth [6]. Therefore, improved screening for the most common CHDs may have consequences on the optimization of pregnancy management. A ventricular septal defect (VSD) is the most common type of CHD. Fetuses with cyanotic CHD have fetal growth restriction, impaired head growth [7]. A variety of CHDs contain VSDs, and the position of the VSD can infer the types of CHD in some cases. Therefore, observation of the ventricular septum is essential for the detection of CHD, which obstetrician and other examiners require high skill levels for its clear depiction in fetal cardiac ultrasound screening.

In recent years, deep learning has been applied to medical image analysis, bringing about a revolutionary impact on the existing diagnostic techniques. Image analysis includes image classification, object detection, and image segmentation. Image classification determines whether an object is in the image or not and is the most straightforward task [8]. On the other hand, object detection roughly indicates an object’s location with a square box if it is in the image [9]. Image segmentation is the process of partitioning a digital image into multiple segments pixel-by-pixel and has been applied to various medical images such as retinal vessel and brain tumor detection [10,11]. The goal of segmentation is to simplify and change the representation of an image into something that is more meaningful and easier to analyze. For segmentation methods using deep learning, fully convolutional networks (FCNs) [12], U-Net [13], SegNet [14], PSPNet (Pyramid Scene Parsing Network) [15], and DeepLab (v1–v3+) [16,17] were developed. Among them, U-net, which is a combination of convolution, deconvolution, and skip-connection, is frequently used for medical image segmentation. Furthermore, DeepLab is a network that features pyramidal pooling modules. In addition, DeepLab v3+ incorporates the encoder–decoder model, uses VGG (Visual Geometry Group) for the encoder part, and is currently the state-of-the-art in the field of general image segmentation. In such a case, when compared with other medical imaging modalities such as computerized tomography (CT), magnetic resonance imaging (MRI), and pathological imaging, a limited number of studies have been conducted wherein deep learning was applied to the ultrasound images [18]. In previous studies, classification was conducted using echocardiography views [19,20], wall motion abnormalities [21], ventricular segmentation on adult echocardiographic images. Ghesu et al. reported the detection and segmentation of the aortic valve from three-dimensional (3D) ultrasound images. With respect to neonatal echocardiography [22], Pereira et al. detected the coarctation of the aorta (CoA) by dividing the fetal cardiac ultrasound images into patches, followed by classification using a neural network; the time-series information of videos was not utilized [23]. Regarding fetal echocardiography, Yasutomi et al. used ultrasound images synthesized with artificial shadows, and then trained the neural network to learn the features of the shadows, which created a noise-resistant neural network [24]. Arnaout et al. reported the segmentation results of the thorax, heart, spine, and each of the four cardiac chambers using U-net [25]. Their targets had a larger part than the ventricular septum, and no time-series information of videos was employed.

**Figure 1:**
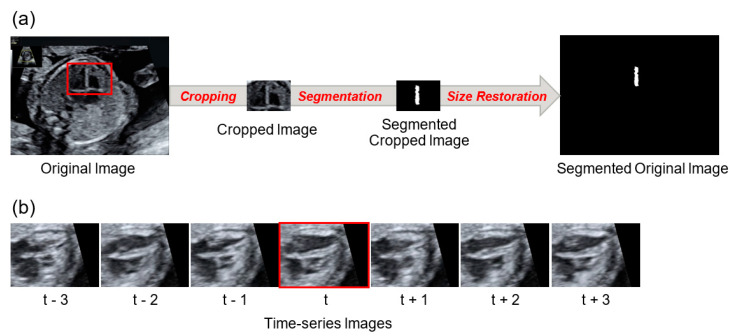
Cropped image and time-series image. (**a**) The “original image” is cropped and transforms into the “cropped image”; the cropped image is segmented and transforms into the “segmented cropped image”. The segmented cropped image is then restored to its original size and transforms into the “segmented original image”. (**b**) A segmentation target image is labelled “t”, and pre-/post- time-series images are labelled “t ± 1, 2, 3”. All of them were cropped.

With respect to the development of ultrasound machine learning technology, our research aims to develop a novel method for image segmentation of ultrasound videos based on deep learning on the four-chamber view, which is one of the standardized transverse scanning planes in fetal cardiac ultrasound screening. The detection of the ventricular septum is challenging, given that a ventricular septum is small and elongated, and its shape changes in accordance with the fetal heartbeat. Furthermore, ultrasound videos contain numerous artifacts, thus increasing the difficulty associated with the accurate determination of the ventricular septum shape. However, experts in fetal cardiac ultrasound screening have overcome these problems using the following methods; (1) by directing significant focus on the area around the ventricular septum and (2) distinguishing noise from actual motion by identifying time-series changes in the ventricular septum from ultrasound videos. Here, we developed a novel method that integrated these expert techniques, namely, the utilization of the cropped image information around the ventricular septum and the time-series information of ultrasound videos (Figure 1). Finally, from a machine learning perspective, we tested the feasibility of our proposed method for normal data without CHD cases. Not intending to get any clinical findings, we investigated the performance comparison of the proposed method and the conventional segmentation methods in this study.

## 2. Materials and Methods

**Figure 2:**
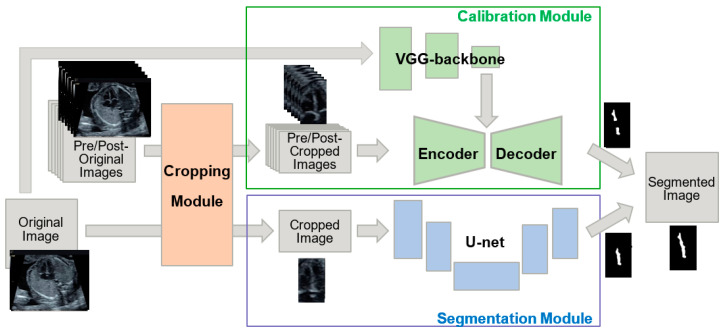
Overview of Cropping-Segmentation-Calibration (CSC). A “cropping module” and “calibration module” were added to improve the U-net-based segmentation results from the “segmentation module”. The cropping module crops out the area around the ventricular septum. Moreover, the calibration module, which consists of an encoder–decoder (ED) and a Visual Geometry Group-backbone module (VGG), calibrated the output of the segmentation module. The ED utilizes time-series information, and VGG utilizes original image information.

In this study, we developed a novel method for segmenting the ventricular septum based on U-net; called Cropping-Segmentation-Calibration (CSC). A key feature of our developed method is the calibration of the results obtained from the U-net. To improve segmentation performance, a “cropping module” and “calibration module” were added to our model. The area around the ventricular septum was excluded from the cropped region using the previously developed YOLO (You Look Only Once) object detection model (Supplementary Figure S1) [8]. The calibration module was employed for the calibration of the segmentation results obtained using the U-net, which uses pre/post-images and the complete image before cropping to process the noise and time-series information specific to ultrasound videos. Figure 2 presents an overview of CSC. In the following sections, a detailed description of each module is presented, in addition to the CSC network, training and inference methods, data, evaluation methods, and comparison methods.

### 2.1. Cropping Module

The objective of the cropping module is to obtain images around the ventricular septum. Supplementary Figure S1 presents an overview of the cropping module. YOLOv2 [26] was employed to detect the bounding box (BBOX) of the ventricular septum. The BBOX contains information about the location (X-coordinate of the center, Y-coordinate of the center, X-width, and Y-width; where (X, Y) are the co-ordinates of the pixel), and the confidence level (real number within the range of 0–1) of the object. The BBOX with the highest confidence level (>0.01), obtained by YOLOv2, is selected, and the image is cropped within the range of the BBOX multiplied by 1.2 (X coordinate of the center, Y coordinate of the center, X-width × 1.2, and Y-width × 1.2). The values are multiplied by 1.2 because the objective of the cropping module is to crop the area around the ventricular septum with minimal leakage and not to determine whether the object is a ventricular septum; although YOLO’s performance is inadequate with an average precision of 0.220, multiplying the BBOX by 1.2 minimizes detection leakage. The cropping of image *Y* according to a BBOX obtained by image *X* with YOLO parameters *θ*_YOLO_ is denoted as follows:

$$\mathrm{Crop}\left(Y,\;X;\;\theta_{\mathrm{YOLO}}\right).$$

**Figure A1:**
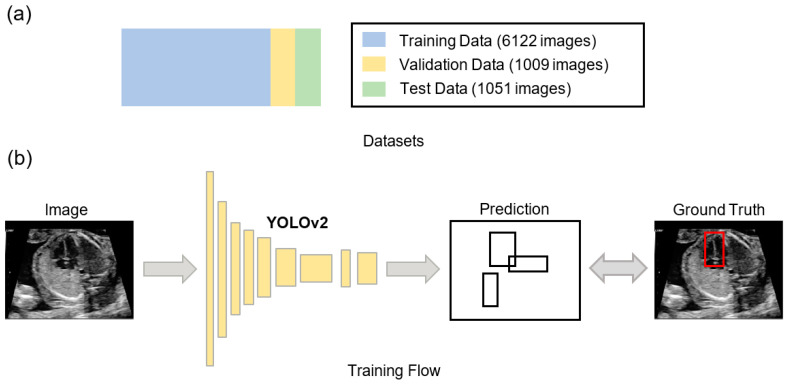
Pretraining for YOLO. (**a**) Datasets for YOLO’s pretraining. The ultrasound images were assigned to 6122 training data, 1009 validation data, and 1051 test data. (**b**) YOLO’s training flow. YOLO was trained by processing the image to calculate the bounding box and comparing it to the ground truth label.

CSC does not induce segmentation without a BBOX with a confidence level of 0.01 or higher. A distinction can be made between a cropped image as the “cropped image” and an image that was size-restored by embedding a cropped image based on BBOX information as the “original image”. The training parameter *θ*_YOLO_ was optimized by ventricular septum-annotated ultrasound images according to the standard training protocol of YOLOv2. The training data and results are shown in Appendix A (Figure A1). In particular, the cropping module was inputted with ultrasound images resized to 416 × 416 pixels, and the output was resized to 256 × 256 pixels. In YOLO, six frames of the video *X*_*t* = ±1,2,3_ at pre/post-times are inputted to calibration module, and the area around the ventricular septum is cropped. The segmentation target images (*X*_*t* = 0_) are also cropped as inputs of the segmentation module (Figure 2).

### 2.2. Segmentation Module

In the segmentation module, an improved version of U-net was introduced in pix2pix. The network configuration is described in the following subsection. We feed a cropped image Crop(*X*_*t* = 0_, *X*_*t* = 0_; *θ_YOLO_*) to the U-net, and the U-net outputs a segmented image of the ventricular septum, as follows:

$$\mathrm{Seg}\left(X_{t\;=\;0};\;\theta_{\mathrm{YOLO}},\theta_{\mathrm{UNET}}\right)=\mathrm{UNET}\left(\mathrm{Crop}\left(X_{t\;=\;0},X_{t\;=\;0};\;\theta_{\mathrm{YOLO}}\right);\theta_{\mathrm{UNET}}\right).$$

It should be noted that each pixel of the segmented image is a real number within the range 0–1, and it denotes the confidence of the segmentation thus performed.

### 2.3. Calibration Module

The calibration module is used to calibrate the results of the segmentation module using two models; namely, the encoder–decoder model (denoted as E and D, respectively; with the synthesis function denoted as ED) and the VGG-backbone model (denoted as VGG). Encoder E is populated with pre/post-images Crop(*X*_*t* = ±1,2,3_, *X*_*t* = ±1,2,3_; *θ*_YOLO_), in which the ventricular septum is cropped by YOLO. Moreover, E(Crop(*X*_*t* = ±1,2,3_, *X*_*t* = ±1,2,3_; *θ*_YOLO_)) carries out the function of embedment, which results in a compressed representation of the pre/post-event time information. The VGG employed was the VGG-16 model [9] without the output layer pretrained using the ImageNet database as the embedment for the original image. The original image *X*_*t* = 0_ is inputted to the VGG, and the embedment of *X*_*t* = 0_ is obtained as VGG(*X*_*t* = 0_; *θ*_VGG_). With these two embedded images as inputs, D outputs the pixel-by-pixel calibration values:

$$\mathrm{Cali}\left(X_{t\;=\;0\;\pm 1,2,3};\;\theta_{\mathrm{YOLO}},\theta_{E},\theta_{D},\theta_{\mathrm{VGG}}\right)=\;D\left(E\left(\mathrm{Crop}\left(X_{t\;=\;\pm 1,2,3},\;X_{t\;=\;\pm 1,2,3};\;\theta_{\mathrm{YOLO}}\right);\theta_{E}\right)\right)||\;\mathrm{VGG}\left(X_{t\;=\;0};\;\theta_{\mathrm{VGG}}\right);\theta_{D}),$$

where “||” denotes the concatenation of the vectors. The output of the calibration module is the segmented image, and each pixel of the segmented image, which is a real number ranging from 0 to 1 that denotes the confidence of segmentation. The output of the segmentation module and the calibration module are integrated for each pixel to produce the final output of the CSC network. Hence, the following is obtained,

$$\mathrm{Seg}\left(X_{t\;=\;0};\;\theta_{\mathrm{YOLO}},\theta_{\mathrm{UNET}}\right)+\mathrm{Cali}\left(X_{t\;=\;0\;\pm 1,2,3};\;\theta_{\mathrm{YOLO}},\theta_{E},\theta_{D},\theta_{\mathrm{VGG}}\right),$$

where “+” denotes element-wise addition.

### 2.4. CSC Network

The cropping module employed YOLOv2, which is based on the CNN “darknet”, and has an output layer that simultaneously outputs the position and confidence of a BBOX (see cropping module section). For the segmentation module, U-net was improved in pix2pix. This U-net is based on a CNN and employs convolutional layers and max-pooling layers for the encoder, in addition to up-convolution for the decoder. The activation function was a rectified linear unit (ReLU), and a 50% dropout was applied to the last layer. The input size was 256 × 256 pixels, and the output was 256 × 256 pixels. Each pixel of the input was a real number ranging from -1 to 1, and each pixel of the output was a real number ranging from 0 to 1 (Supplementary Figure S2a). For the calibration module, the encoder employed a convolutional layer and max spooling, the decoder employed up-convolution; and the activation function was ReLU. The VGG employed an ImageNet-trained VGG-16 network, with the exception of the final layer. The input size was 224 × 224 pixels, and the output was 2048 pixels. The network of calibration modules is detailed in Supplementary Figure S2b.

### 2.5. Training Procedure

The training was divided into Phase 1 and Phase 2 (Supplementary Figure S3a,b). In Training phase 1, the embedding of pre/post-images was trained first (*θ*_E_), followed by the training of the decoder part (*θ*_D_) of the ED and U-net (*θ*_UNET_) in Training phase 2. Annotated images of the ventricular septum *Y*_*t* = 0_, were used as ground truth labels in both phases; ground truth labels is another name for answer labels. Moreover, *θ*_YOLO_ was trained using the dataset (Appendix A) based on *θ*_VGG_ ImageNet.

In Training phase 1, the decoder was defined as $\;\hat{D}$, which was the same as D, with the exception that the output was six images. Moreover, the training parameters of the encoder (*θ*_E_) and those of the decoder (*θ*${}_{\hat{D}}$) were trained to output exactly the same images as the input images Crop(*X*_*t* = *±*1,2,3_, *X*_*t* = ±1,2,3_; *θ*_YOLO_) by inputting the cropped pre/post-time images of the training data into the ED (Supplementary Figure S3a). Binary cross-entropy was used for the loss function:

$$\underset{\theta_{E},\;\;\;\theta_{\;\hat{D}}}{\mathrm{Minimize}\;\mathrm{Loss}}\;\left(\mathrm{Crop}\left(X_{t\;=\;\pm 1,\;2,3},\;X_{t\;=\;\pm 1,2,3};\;\theta_{\mathrm{YOLO}}\right),\;\hat{D}\left(E\left(\mathrm{Crop}\left(X_{t\;=\;\pm 1,2,3},\;X_{t\;=\;\pm 1,2,3};\theta_{\mathrm{YOLO}}\right);\;\theta_{E}\right);\theta_{\;\hat{D}}\right)\right).\;$$

In Training phase 2, Decoder $\;\hat{D}$ was replaced by Decoder D. The decoder part (*θ*_D_) of the ED and the U-net (*θ*_UNET_) were then trained to achieve the maximum agreement between the integrated images (output image of segmentation module and calibration module) and the ground truth labels (module) (Supplementary Figure S3b). Hence, Phase 2 training can be described as follows:

$$\begin{array}{c} \underset{\theta_{\mathrm{UNET}},\;\;\theta_{D}}{\mathrm{Minimize}\;\mathrm{Loss}}\;(\mathrm{Crop}(Y_{t\;=\;0},\;X_{t\;=\;0};\;\theta_{\mathrm{YOLO}}),\;\mathrm{Seg}\left(X_{t\;=\;0};\;\theta_{\mathrm{YOLO}},\theta_{\mathrm{UNET}}\right)\\ +\mathrm{Cali}\left(X_{t\;=\;0\;\pm 1,2,3};\;\theta_{\mathrm{YOLO}},\theta_{E},\theta_{D},\theta_{\mathrm{VGG}}\right)), \end{array}$$

where the loss function was set as the per-pixel binary-cross entropy of the difference between the output image and the ground truth label. 

Phases 1 and 2 of training were conducted with an epoch of 200 and batch size of 12, and a model was outputted in intervals of five epochs. The Adam optimizer was employed, and the learning rate was 0.001. Among the output models, the model with the lowest loss in the validation data was used for inference. 

### 2.6. Data Preparation

#### 2.6.1. Data Acquisition Method

In this study, we used 421 fetal cardiac ultrasound videos of 211 pregnant women who were screened in the second trimester. The videos were captured at four Showa University Hospitals (Tokyo and Yokohama, Japan). All women were enrolled in research protocols approved by the Institutional Review Board of RIKEN, Fujitsu Ltd., Showa University, and the National Cancer Center (approval identification number: Wako1 29-4). In addition, all methods were carried out in accordance with the Ethical Guidelines for Medical and Health Research Involving Human Subjects; with respect to the handling of data, the Data Handling Guidelines for the Medical AI project were followed.

Not only expert sonographers but also obstetricians with at least three years of experience obtained ultrasound videos under the guidance of experts. Each video consisted of the sequential cross-sections from the base of the heart to the vascular arches. Ultrasound videos of only normal cases diagnosed by expert sonographers were used, which exhibited no observable structural abnormalities in the fetuses. After birth, the pediatrician checked vitals and heartbeats daily for the first five to seven days. In addition, the pediatrician performed a one-month postnatal check-up and all cases were finally confirmed as normal. The videos were captured using ultrasonography machines (Voluson^®^ E8 or E10, GE Healthcare, Chicago, IL, USA) equipped with an abdominal 2–6 MHz transducer. A cardiac preset was used, and images were magnified until the chest fills at least one half to two-thirds of the screen, with an ultrasonic frequency of 40 Hz. In particular, 615 images with a four-chamber view, which included the ventricular septum, were selected irrespective of the axis orientation and ventricular systolic/diastolic bias. Moreover, the set of three pre/post-frames in the video of the selected images were extracted. Both the systolic and diastolic images were included in the segmentation target images and pre/post-images. Given that the frame rate was 40 fps and the fetal heart rate in the second trimester ranged from 140 to 160 bpm, the images at ±3, 6, and 9 frames were extracted around the target image.

#### 2.6.2. Data Preprocessing

The ventricular septa in the original images were annotated pixel-by-pixel, and the obstetricians created the correct answer labels. No annotations were set for the pre/post-images; they were only selected. The images and labels were 640 × 480 pixels, and the format was unified to the Portable Networks Graphic (PNG) format. The dataset was split into training data and test data in a ratio of 2:1. One-fifth of the training data were used as validation data. To perform three-fold cross-validation, three datasets with different combinations of training and test data were prepared (Supplementary Figure S4).

### 2.7. Metrics

The statistical indexes, intersection over union (IoU), and Dice coefficient (Dice) are generally used to quantify the performance of image segmentation methods. When true-positive pixels are defined as TP, false-negative pixels as FN, and false-positive pixels as FP, these indexes are calculated as follows:

$$\mathrm{IoU}=\frac{\mathrm{TP}}{\mathrm{TP}+\mathrm{FP}+\mathrm{FN}}$$

$$\mathrm{Dice}=\frac{2\mathrm{TP}}{2\mathrm{TP}+\mathrm{FP}+\mathrm{FN}}.$$

For the inference results and correct labels, the IoU for each image was considered, and the mean across all images was calculated; namely, the mean intersection over union (mIoU). In addition, the Dice per image were considered, and the mean across all images was calculated; namely, the mean Dice coefficient (mDice). To calculate these metrics, a confidence level of 0.5 was employed as the threshold value for each pixel of the segmented image. In this study, three-fold cross-validation was conducted, and the mIoU and mDice of the three datasets were calculated. The standard deviation was calculated. In particular, the cropped images and original images were evaluated (Figure 1). The IoU and Dice were calculated as 0 for each image in which YOLO failed to detect the ventricular septum.

### 2.8. Experiments and Comparison

First, the performance of the segmentation method DeepLab v3+ was compared with CSC. This is the highest performing segmentation method in general imaging using the original U-net. The settings of DeepLab v3+ were 30,000 iterations and four batches. Moreover, the image size was 513 × 513 pixels, the back-born network was VGG, and data augmentation was not applied. Stochastic gradient descent with Nesterov momentum was employed with a momentum of 0.9, initial learning rate of 0.007, decay rate of 0.9 per 2000 iterations. The source code of DeepLab v3+ is available at https://github.com/mathildor/DeepLab-v3. Furthermore, the impact of each module on performance due to variations in the combination of YOLO, ED, and VGG was evaluated (Supplementary Figure S5). Finally, to determine whether the segmentation accuracy is influenced by the orientation of the heart axis and ventricular systole/dilation, the performances of the varied combinations of the YOLO, ED, and VGG were compared. To align the conditions, data augmentation was not conducted.

All original codes are available at https://github.com/rafcc/2020-prenatal-vs-segmentation, which is implemented in Keras ver2.2 backend of TensorFlow ver1.12. All the experiments were carried out using a computer cluster with an Intel(R) Xeon(R) central processing unit (CPU) E5-2690 v4 at 2.60 GHz, GeForce GTX 1080 Ti.

## 3. Results

### 3.1. Data Characteristics

The median number of gestational weeks for the 211 pregnant women enrolled was 20 weeks (range: 18–28 weeks) (Supplementary Figure S6). A total of 615 normal fetal cardiac ultrasound images were selected from 421 ultrasound videos. The angle of insonation of VS changed little throughout the ultrasound video. The mean and standard deviation of the angle between the ventricular septum orientation and the ultrasound beam entry were 49° and 36°, respectively. The minimum angle was 0°, and the maximum angle was 174°. This dataset was classified by the cardiac axis orientations, which were divided into 297 images as apical, and 318 images as non-apical; we defined apical as the ventricular septum orientation within ±30 degrees when the vertical direction was set to 0 degrees, and non-apical as anything else. Moreover, when classified by the ventricular systolic state, the dataset was divided into 301 images classified as systole and 314 classified as diastole (Table 1).

### 3.2. Comparison with the Existing Methods

**Figure 3:**
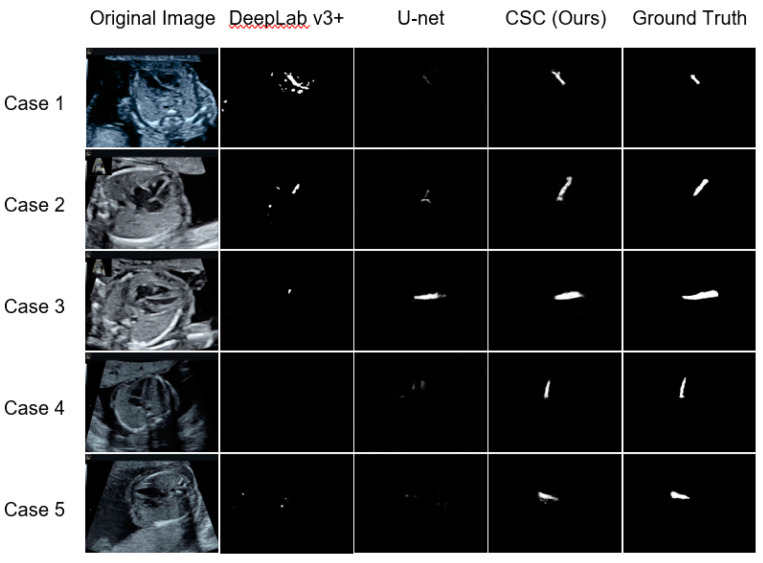
Representative examples of the ventricular septum segmentation images in test data for the existing methods (DeepLab v3+ and U-net) and CSC (Cropping-Segmentation-Calibration). One horizontal row presents the segmentation results with respect to each method for the same case. The white pixels are estimated as the ventricular septum, and the degree of whiteness indicates the confidence level. Among the three methods, the segmentation results of CSC were most in accordance with the ground truth.

First, Figure 3 presents a comparison of the segmentation results of CSC with those of DeepLab v3+ and the original U-net. CSC detected the ventricular septum in most of the images; whereas, using the other methods, the ventricular septum was detected in only a few images.

A discussion is presented on the effect of cropping using numerical metrics. Table 2 presents the results of the mIoU and mDice calculated for each method. The mIoU and mDice calculated from the segmentation results and ground truth labels were 0.5543 and 0.6891, respectively; the segmentation from the cropped images was slightly improved.

Third, a discussion is presented on the difference between CSC and existing methods. The mIoU (mDice) was 0.0224 (0.0382), 0.1519 (0.2238), and 0.5543 (0.6891) for DeepLab v3+, the original U-net, and CSC, respectively. CSC yielded the highest value. From a comparison between DeepLab v3+ and the original U-net with respect to medical imaging, several studies reported that DeepLab was superior to U-net [27]; whereas, other studies reported otherwise [28]. In this study, U-net exhibited superior performance to DeepLab.

### 3.3. Comparison of Modules

**Figure 4:**
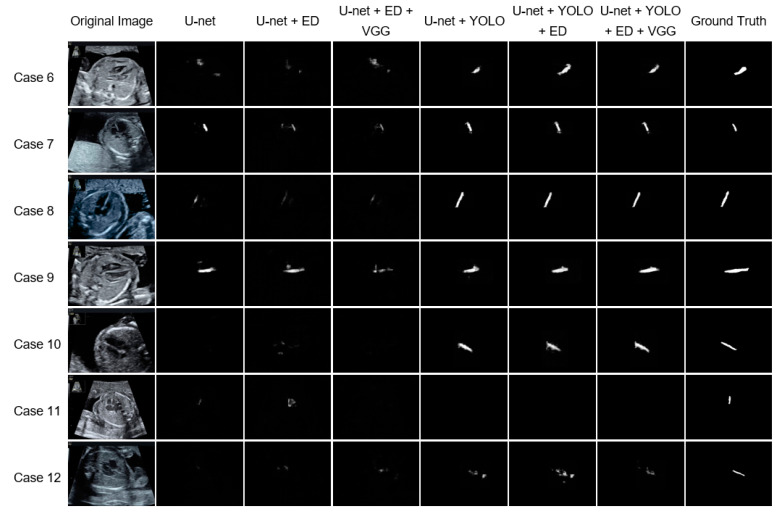
Representative examples of the ventricular septum segmentation in test data for each module combination. One horizontal row presents the segmentation results obtained using each method for the same case. The white pixels are estimated as the ventricular septum, and the degree of whiteness indicates the confidence level. Among the various module combinations, the segmentation results of U-net + YOLO (You Look Only Once) + ED + VGG (CSC) were most in accordance with the ground truth. The YOLO significantly contributed to the improvement of the segmentation accuracy, and ED improved the segmentation. Moreover, the addition of VGG slightly narrowed the prediction section.

To evaluate the effectiveness of the YOLO, ED, and VGG modules, different combinations of modules were tested (Figure 4). The segmentation images revealed that the accuracy improved notably using YOLO. When ED was added to YOLO, the detection range increased; when VGG was added, a portion of the overspread pixels was excluded.

Table 3 presents the mIoU and mDice for each module combination involving YOLO. For the three combinations of U-net + YOLO, U-net + YOLO + ED, and U-net + YOLO + ED + VGG; the mIoU (mDice) of the cropped and the original images were 0.5424 (0.6782)/0.5373 (0.6724), 0.5587 (0.6944)/0.5533 (0.6885), and 0.5598 (0.6950)/0.5543 (0.6891), respectively. For each combination, the mIoU and mDice were slightly higher before restoration to the original image size than after restoration.

From a comparison of the combinations with and without YOLO, the mIoU and mDice increased significantly for those with YOLO. For each of the three patterns, namely, U-net, U-net + ED, and U-net + ED + VGG, the mIoU (mDice) with and without YOLO were 0.1519 (0.2238)/0.5373 (0.6724), 0.0633 (0.0996)/0.5533 (0.6885), and 0.0902 (0.1400)/0.5543 (0.6891), respectively. Although the cropping module was set to increase recall, several images could not be cropped. Among the 615 ultrasound images, YOLO failed to detect the ventricular septum in four images, irrespective of whether the ventricular septum was included; these four images were also not segmented. As shown in Case 11 in Figure 4, the ventricular septum was not included in the 13 cropped images. Although there were several images in which part of the septum was excluded during cropping, the segmentation accuracy increased due to the YOLO cropping.

The addition of ED to YOLO increased the mIoU (mDice) from 0.5373 (0.6724) to 0.5533 (0.6885). When VGG was added, mIoU (mDice) increased from 0.5533 (0.6885) to 0.5543 (0.6891), thus exhibiting a slight upward trend. In contrast, without YOLO cropping, the mIoU (mDice) decreased from 0.1519 (0.2238) to 0.0633 (0.0996) when ED was added. When VGG was added, the mIoU (mDice) increased from 0.0633 (0.0996) to 0.0902 (0.1400), thus indicating an improvement; however, that of the original U-net was still higher.

### 3.4. Effects of Cardiac Axis Orientation and Ventricular Systolic State

**Figure 5:**
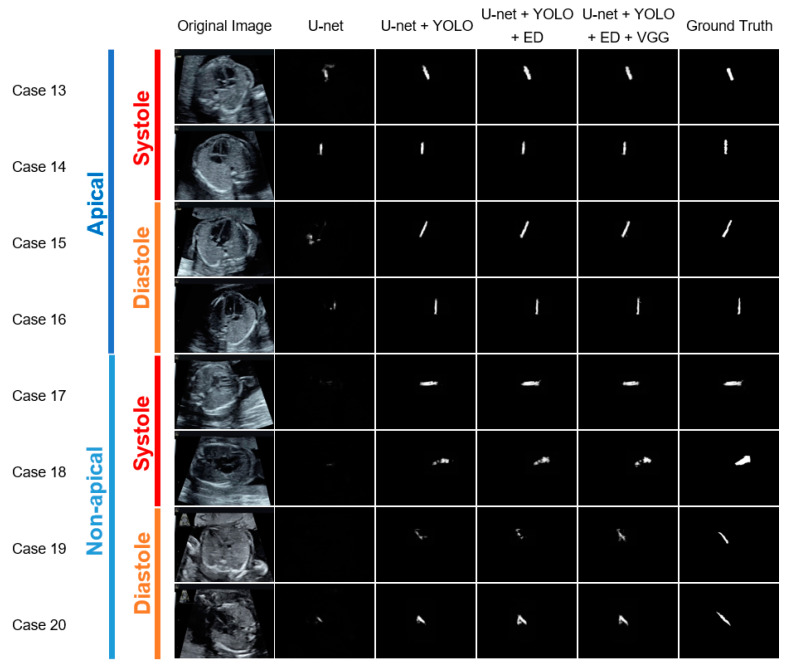
Representative examples of the ventricular septum segmentation images classified by the cardiac axis orientation and ventricular systolic state, from the test data, for each module combination. One horizontal row presents the segmentation results obtained using each method for the same case. The white pixels are estimated as the ventricular septum, and the whiteness indicates the confidence level. The segmentation results were more accurate for the apical group than for the non-apical group, and more accurate for the diastolic group than for the systolic group, irrespective of the module combination. The addition of the YOLO significantly improved the segmentation accuracy, and the addition of the ED further improved it, irrespective of cardiac axis orientation and ventricular systolic state. Moreover, the addition of VGG slightly improved the segmentation accuracy for the systolic and non-apical groups.

To assess the influence of the cardiac axis orientation and ventricular systolic state on segmentation accuracy, the segmentation images of the test data for each combination of modules was classified according to the cardiac axis orientation (apical group vs. non-apical group) and ventricular systolic state (systolic group vs. diastolic group) (Figure 5). We defined apical as the ventricular septum orientation within ± 30 degrees when the vertical direction was set to 0 degrees, and non-apical as anything else.

The mIoU and mDice were calculated for each group (Table 4 and Table 5). The segmentation accuracy was higher for the apical group than for the non-apical group, and higher for the diastolic group than for the systolic group, irrespective of the module combination. CSC yielded the highest value in the non-apical group. In particular, the mIoU (mDice) for the non-apical group with respect to CSC was 0.5255 (0.6688). However, in the apical group, U-net + YOLO + ED yielded the highest value, in that the mIoU (mDice) was 0.5889 (0.7146). From the comparison between the diastolic and systolic groups, CSC yielded the highest value in the systolic group, in that the mIoU (mDice) was 0.5435 (0.6755). However, in the diastolic group, U-net + YOLO + ED yielded the highest value, in that the mIoU (mDice) was 0.5655 (0.7037). 

The combination of U-net and YOLO significantly improved the segmentation accuracy, and the further addition of ED slightly improved the accuracy; irrespective of the cardiac axis orientation and ventricular systolic state. However, when VGG was added, there was a slight decrease in the accuracy in the apical and diastolic groups. Both groups exhibited relatively high accuracies without the addition of VGG. Moreover, the non-apical and systolic groups yielded relatively low accuracies, which slightly increased after the addition of VGG.

## 4. Discussion

The importance of fetal cardiac ultrasound screening has recently been promoted to improve the prenatal detection rate and provide effective treatment for CHDs [29]. As major CHDs often contain VSDs and the ventricular septum is an important site for determining the cardiac axis and position, a detailed observation of the ventricular septum is essential. However, it is challenging to accurately detect the ventricular septum from ultrasound videos given the numerous artifacts and non-uniform image quality. To overcome these issues and facilitate ventricular septum detection, we proposed a novel segmentation method that is specific to the ventricular septum in ultrasound videos. With respect to the video segmentation, in several studies, the time-series information and object detection techniques were used [30,31]. However, fetal cardiac ultrasound is not an appropriate target for conventional time-series segmentation methods using optical flow, due to the rapid variations in the shape of the heart, in addition to the emergence and disappearance of various substructures of the heart in ultrasound videos. Yu et al. employed cropped images and time-series information to segment the left ventricle of a fetal heart; however, the target was larger than the ventricular septum, and the cropping around the target was performed manually [32]. With respect to the diagnostic techniques of the experts in fetal cardiac ultrasound screening, CSC employs the cropped image information around the ventricular septum and the time-series information of ultrasound videos. In this study, the number of annotated images was small in comparison to previous reports of segmentation on fetal echocardiographic images [22,31]. To overcome this issue and validate the accuracy of the method, we employed six times as many unannotated time-series images of the comparable number of cases.

Compared with the existing segmentation methods, namely, DeepLab v3+ and the original U-net, the ventricular septum detection accuracy was significantly improved by CSC, both visually and numerically, and this method is useful for the detection of the detailed structures of the heart from fetal ultrasound images. From a comparison of the mIoU and mDice for the cropped and original images segmented by CSC, both values were slightly higher for the cropped images. It is highly probable that this was because, in several images, portions of the ventricular septum were excluded due to cropping, and the excluded pixels were calculated as FN in the original size image, which resulted in the reduction of the IoU and Dice. 

Finally, we compared the calculation time. For one CPU and one GPU (see Section 2.8 for details), the CSC computation time was 0.1343 s, with a standard deviation of 0.1023 s per image. For comparison, the computation time for U-net was an average of 0.0556 s and a standard deviation of 0.0027 s per image. CSC achieved more than three times the performance improvement in about three times the computation time of U-net. Thus, CSC is considered to be sufficiently practical to support examiners in fetal cardiac ultrasound screening.

### 4.1. Combination Analysis of Modules

Among YOLO, ED, and VGG added to the U-net in CSC, YOLO was found to be the most effective in improving the accuracy. With respect to general images, it was suggested in several studies that the integration of object detection and segmentation, i.e., instance segmentation, is useful. When the size of the object to be segmented is small, as is the case with the ventricular septum, it is useful to target the segmentation area by cropping, as this reduces the risk of detecting the wrong area.

Given that the mIoU and mDice were increased by the addition of ED to YOLO, the video time-series information was considered as useful for the segmentation of the ultrasound images. Figure 4 (Cases 6, 7, 10, and 12) shows that the segmentation section expanded to compensate for the noise upon the addition of ED. Without YOLO cropping, the addition of time-series information by ED decreased the mIoU and mDice, thus indicating that YOLO is essential when employing time-series information. In addition, the addition of pre- and post-timing images may allow for the more unnecessary information to be incorporated, instead of the necessary information. With respect to the U-net + ED results for Cases 7, 10, 11, and 12, as shown in Figure 4, the addition of ED decreased the prediction accuracy; thus, it was difficult to identify the ventricular septum. When VGG was added to U-net + YOLO + ED, an improvement in the accuracy was expected by the incorporation of information from the complete image before cropping. However, a slight upward trend was observed. On the basis of the segmentation images, VGG fine-tuned and reduced the number of pixels that were over-extended by the ED.

### 4.2. Heart Axis and Ventricular Systole

When comparing the segmentation accuracy with respect to the cardiac axis orientation, the accuracy was higher in the apical group than in the non-apical group. It is highly probable that this was because the segmentation images underestimated the ventricular septum. Therefore, the larger segmentation of the ventricular septum in the images resulted in a larger FN, and therefore a larger denominator in the IoU and Dice formulas, thus yielding lower values. Accordingly, it was assumed that the slightly higher mIoU and mDice in the apical group was due to the elongated ventricular septum and narrower detection target of the apical group. For more detailed explanation, see Appendix B. Similarly, the mIoU and mDice were slightly higher for the diastolic group than the systolic group, which can be attributed to the thicker septum, and the target to be detected was larger in the systolic group. The addition of VGG with respect to the non-apical and systolic groups slightly increased the mIoU and mDice; thus, the VGG may slightly compensate for the difference of the object.

### 4.3. Limitations

There are several limitations in this study. First, only four-chamber view images were used in this study. The ventricular septum has a different appearance from other views, such as the short-axis view, long-axis view, and five-chamber view. To detect the ventricular septum from a particular view, we need more training data from various views. Second, the examiners captured fetal cardiac ultrasound videos in the dataset using the same type of ultrasonography machine and typical settings for fetal cardiac ultrasound screening. Hence, the reproducibility of applying CSC to other videos, which general examiners take with different equipment and settings, is unknown and needs to be verified in future work. Third, the implementation of CSC requires a GPU-equipped PC so that it comes at a certain cost. Depending on the performance of GPU and the method of implementation, the algorithm may be time-consuming. Finally, we only employed normal data in this study and did not employ abnormal data such as VSDs. Therefore, CSC is a method for precisely detecting the normal ventricular septum and not for detecting abnormalities. Additionally, we did not compare CSC with manual operations of clinicians in the accuracy of detecting the ventricular septum. Thus, CSC has shown progress as a segmentation method in ultrasound videos; however, it is not directly applicable to clinical applications in the present form.

## 5. Conclusions

In this study, a novel method was developed for the precise segmentation of the ventricular septum in fetal cardiac ultrasound videos using the cropped and original image information, in addition to the time-series information. CSC showed a considerable improvement in segmentation accuracy comparing with other representative segmentation methods. For future work, the next step is to validate algorism using normal and abnormal data in experimental settings. The final goal is the automatic detection of ventricular septal abnormalities in clinical settings. Another direction is the automatic measurement of cardiac indexes, such as the cardiac axis.

## Figures and Tables

**Table 1:** Distribution of test data classified by cardiac axis orientation and ventricular systolic state.

Ventricular Systolic State	Apical	Non-Apical	Total
Systole	183	118	301
Diastole	114	200	314
Total	297	318	615

**Table 2:** Evaluation of segmentation results obtained using existing methods (DeepLab v3+ and U-net) and CSC (Cropping-Segmentation-Calibration) with respect to the mIoU and mean Dice coefficient (mDice).

Method	mIoU		mDice	
	Original Image	Cropped Image	Original Image	Cropped Image
DeepLab v3+	0.0224 ± 0.0085		0.0382 ± 0.0140	
U-net	0.1519 ± 0.0596		0.2238 ± 0.0777	
CSC (Ours)	0.5543 ± 0.0081	0.5598 ± 0.0067	0.6891 ± 0.0104	0.6950 ± 0.0074

The values are the mean ± standard deviation of the three datasets for cross-validation. CSC yielded the highest values. Moreover, the cropped images yielded slightly higher values than the original images in CSC.

**Table 3:** Evaluation of segmentation results for each combination of modules with respect to the mIoU and mDice.

U-Net	YOLO	ED	VGG	mIoU		mDice	
				Original Image	Cropped Image	Original Image	Cropped Image
✓				0.1519 ± 0.0596		0.2238 ± 0.0777	
✓		✓		0.0633 ± 0.0372		0.0996 ± 0.0538	
✓		✓	✓	0.0902 ± 0.0304		0.1400 ± 0.0442	
✓	✓			0.5373 ± 0.0134	0.5424 ± 0.0107	0.6724 ± 0.0188	0.6782 ± 0.0153
✓	✓	✓		0.5533 ± 0.0139	0.5587 ± 0.0138	0.6885 ± 0.0141	0.6944 ± 0.0123
✓	✓	✓	✓	0.5543 ± 0.0081	0.5598 ± 0.0067	0.6891 ± 0.0104	0.6950 ± 0.0074

The values are the mean ± standard deviation of the three datasets for cross-validation. CSC with the combination of all modules yielded the highest values. Moreover, YOLO significantly contributed to the improvement of segmentation accuracy. The cropped images yielded slightly higher values than the original images for all module combinations, including the YOLO. With YOLO, the addition of the ED improved the segmentation accuracy. In contrast, without YOLO, the addition of ED decreased the segmentation accuracy. Upon the addition of VGG, a slight upward trend was observed.

**Table 4:** Segmentation evaluation by mIoU and mDice for each module combination when divided by the orientation of the heart axis.

U-Net	YOLO	ED	VGG	mIoU		mDice	
				Apical	Non-Apical	Apical	Non-Apical
✓				0.1878 ±0.1097	0.1213 ± 0.0186	0.2697 ± 0.1410	0.1845 ± 0.0261
✓	✓			0.5793 ± 0.0315	0.4990 ± 0.0058	0.7064 ± 0.0405	0.6417 ± 0.0086
✓	✓	✓		0.5889 ± 0.0265	0.5210 ± 0.0160	0.7146 ± 0.0351	0.6653 ± 0.0140
✓	✓	✓	✓	0.5855 ± 0.0167	0.5255 ± 0.0016	0.7114 ± 0.0264	0.6688 ± 0.0026

The values are the mean ± standard deviation of the three datasets for cross-validation. The apical group yielded higher values than the non-apical group. The addition of the YOLO significantly improved the segmentation accuracy, and the addition of the ED further improved it, irrespective of the cardiac axis orientation. The addition of the VGG contributed to the higher values in the apical group.

**Table 5:** Segmentation evaluation by mIoU and mDice for each combination of modules when divided by the ventricular systolic state.

U-Net	YOLO	ED	VGG	mIoU		mDice	
				Systole	Diastole	Systole	Diastole
✓				0.1397 ± 0.0686	0.1631 ± 0.0528	0.2072 ± 0.0914	0.2388 ± 0.0677
✓	✓			0.5255 ± 0.0158	0.5491 ± 0.0114	0.6567 ± 0.0235	0.6882 ± 0.0146
✓	✓	✓		0.5413 ± 0.0196	0.5655 ± 0.0065	0.6733 ± 0.0186	0.7037 ± 0.0067
✓	✓	✓	✓	0.5435 ± 0.0102	0.5648 ± 0.0073	0.6755 ± 0.0127	0.7026 ± 0.0073

The values are the mean ± standard deviation of the three datasets for cross-validation. The diastolic group yielded higher values than the systolic group. The addition of the YOLO significantly improved the segmentation accuracy, and the addition of the ED further improved it, irrespective of the ventricular systolic state. The addition of the VGG contributed to the higher values in the systolic group.

## Supplementary Materials

The following are available online at https://www.mdpi.com/2218-273X/10/11/1526/s1, Figure S1: Overview of the cropping module, Figure S2: Details of network architecture, Figure S3: Flow diagrams of training phase, Figure S4: Training/test split and cross validation, Figure S5: Combinations of each module, Figure S6: Histogram of gestational weeks.

## Appendix A

Datasets and results of YOLOv2: Figure A1 describes YOLO’s pretraining. 6122 normal ultrasound images were employed for the training data, 1051 images for the test data, and 1009 images for the validation data. Among the 6122 images in the training data, 1083 were positive data containing the ventricular septum, and 5035 images were negative data without the ventricular septum. Among the 1051 images in the test data, 247 were positive data and 804 were negative data. Among the 1009 images in the validation data, 351 were positive data and 658 were negative data. This dataset was independently produced from the same video dataset of this study. The average precision of the ventricular septum detection was 0.220 (IoU = 0.5) for the test data, whereas it was 0.376 (IoU = 0.5) for the validation data. Moreover, YOLO successfully detected the ventricular septum; however, its specific location was not identified.

## Appendix B

In this appendix, we further discuss why CSC performance of the apical group is higher than that of the non-apical group. This is because the task settings for image segmentation make the non-apical group a more difficult problem to solve than the apical group.

For the ground truth label in Figure 5, the apical group showed little change (Figure 5, cases 13–16), but the non-apical group showed a large change (Figure 5, cases 18–20). This is because the ultrasound reflects more easily in the non-apical group than in the apical group. Therefore, the changes in the shape of the ventricular septum are more easily captured in the non-apical group. In other words, the non-apical group had more variation in ground truth labels than the apical group.

Thus, the algorithm needs to capture more variation in the non-apical group than in the apical group if ventricular septal segmentation is considered as a task of image segmentation. Hence, it is more difficult for a machine learning algorithm to segment accurately in the non-apical group than in the apical group. This does not mean that the apical group is easier for humans to observe than the non-apical group. In fact, not only for CSC but also for U-net, the performance of the apical group is higher than that of the non-apical group. This indicates that segmentation in the non-apical group is more difficult than in the apical group and this is not a CSC-specific characteristic.
