# Supplementary material for: Image Segmentation of the Ventricular Septum in Fetal Cardiac Ultrasound Videos Based on Deep Learning Using Time-Series Information
Source: Biomolecules. 2020 Nov 8;10(11):1526. doi: 10.3390/biom10111526 (PMC7695246; doi:10.3390/biom10111526)
Supplement: Supplementary file 1 [file biomolecules-10-01526-s001.pdf]

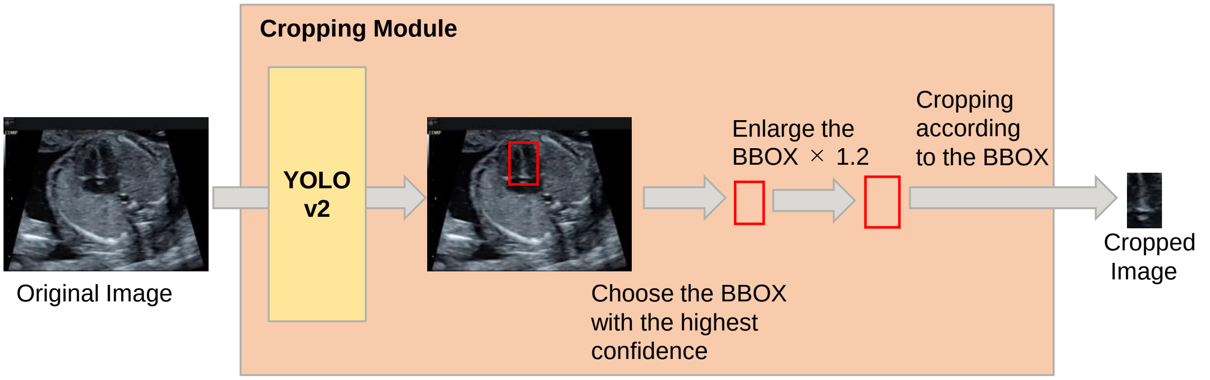

Supplementary Figure S1. Overview of the cropping module. First, the YOLO detects the bounding box (BBOX) of the ventricular septum in the original image. Thereafter, the BBOX with the highest confidence level (> 0.01) is selected. Finally, the image is cropped within the range of the BBOX multiplied by 1.2.

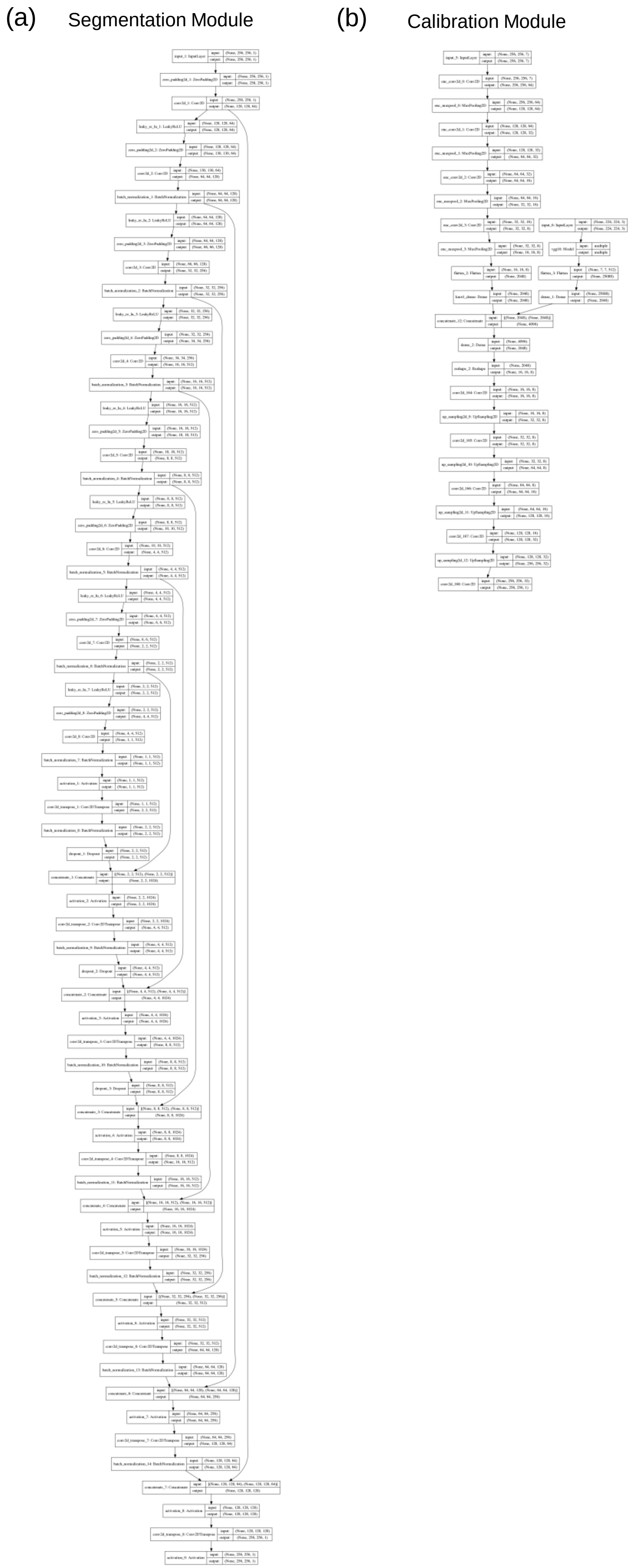

Supplementary Figure S2. Details of network architecture. (**a**) Segmentation module and (**b**) calibration module.

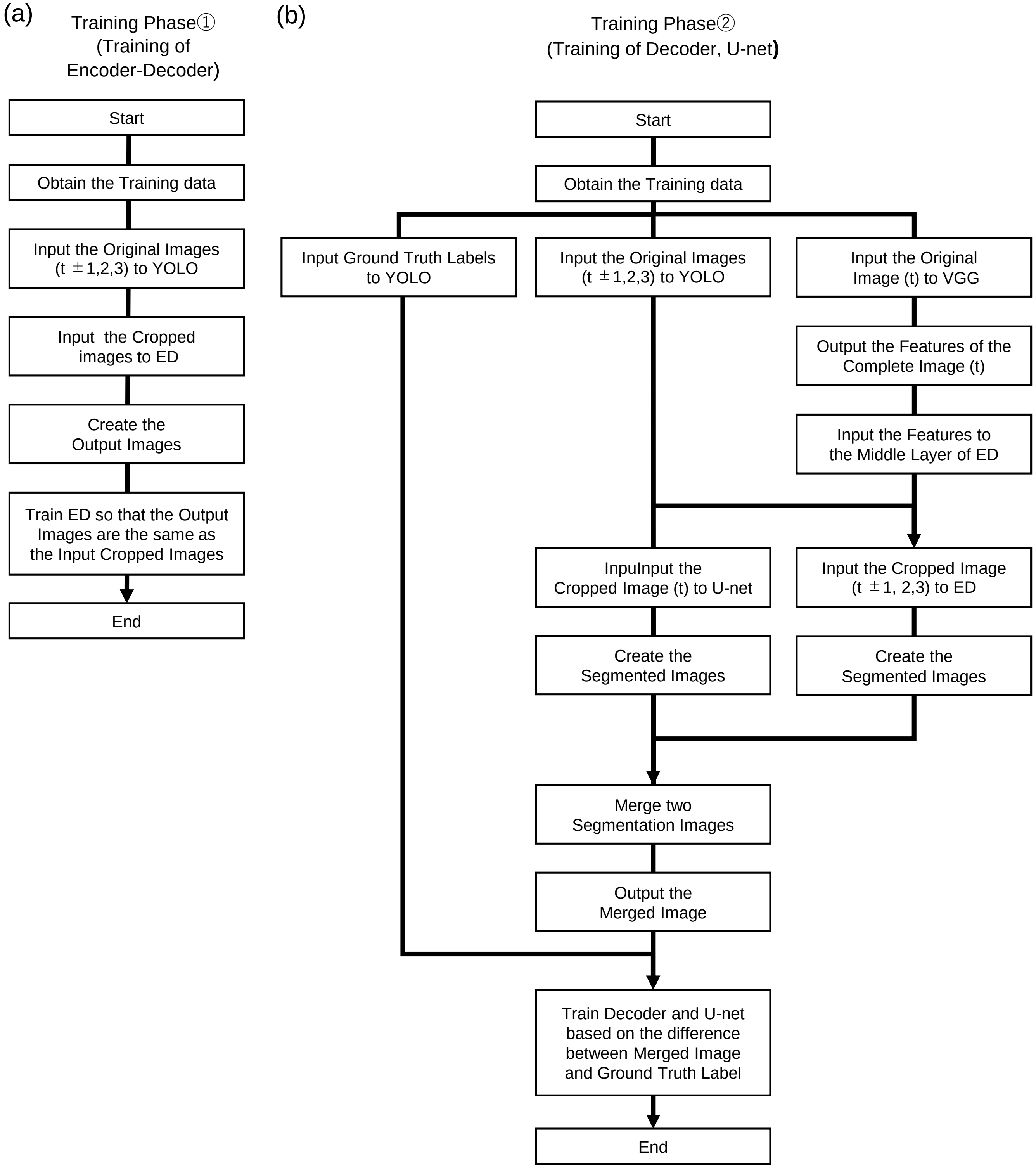

Supplementary Figure S3. Flow diagrams of training phase. (**a**) In Training phase 1, the encoder-decoder (ED) was trained to output the same images as the input (the cropped time-series images). (**b**) In Training phase 2, the decoder part of ED and the U-net were trained to realize the maximum agreement between the merged segmentation images and the ground truth labels by utilizing the cropped time-series information and the features of the original images.

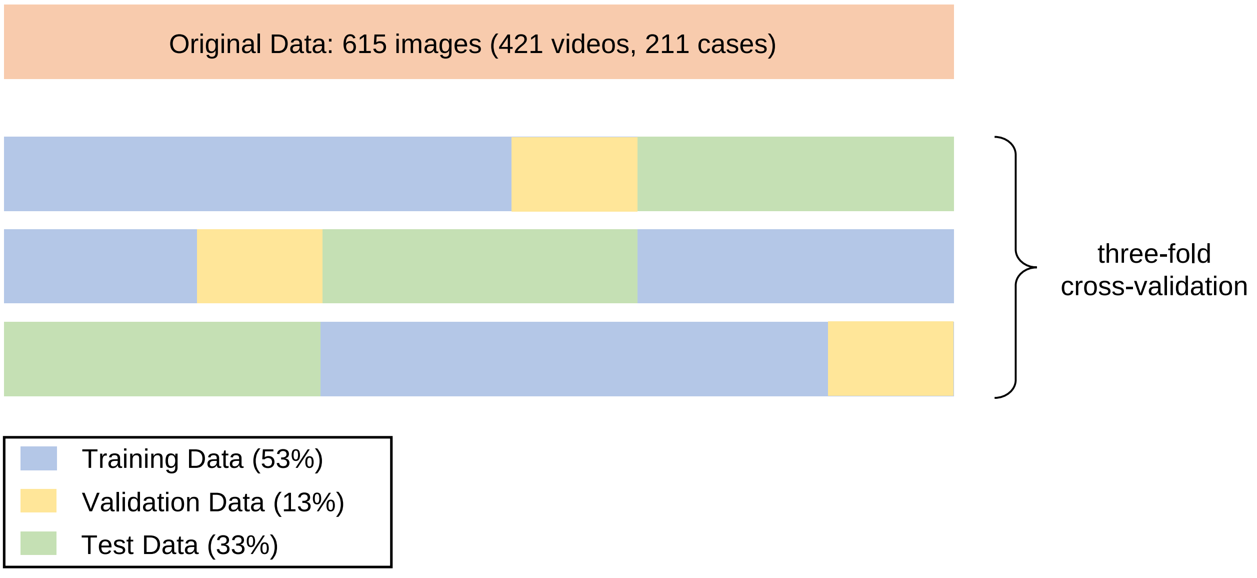

Supplementary Figure S4. Training/test split and cross validation. Six hundred and fifteen images from 421 normal fetal cardiac ultrasound videos were employed, which were extracted from 211 cases. The dataset was assigned a training to test data ratio of 2:1, and one-fifth of the training data was used as validation data. Three-fold cross-validation was performed with different combinations of training and test data.

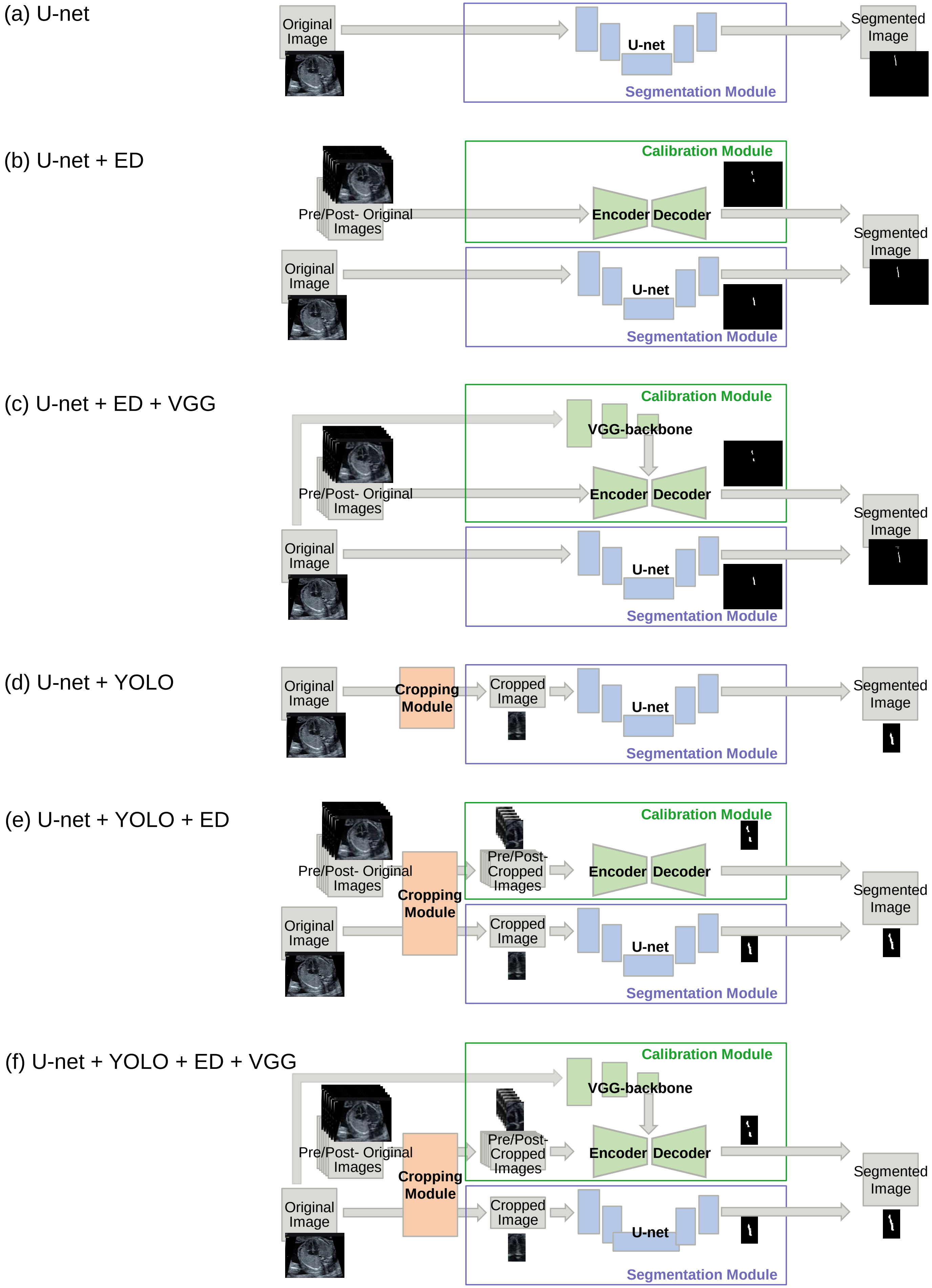

Supplementary Figure S5. Combinations of each module. (**a**) Original U-net only, (**b**) U-net + ED, (**c**) U-net + ED + VGG, (**d**) U-net + YOLO, (**e**) U-net + YOLO + ED, (**f**) U-net + YOLO + ED + VGG (CSC).

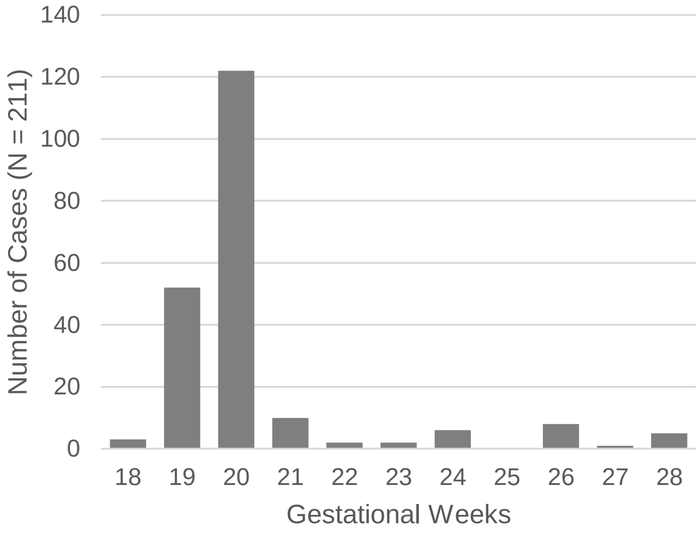

Supplementary Figure S6. Histogram of gestational weeks at the time of ultrasound video acquisition. The median number of gestational weeks for the 211 pregnant women enrolled was 20 weeks (range: 18–28 weeks).
